# Case report of a double-wave re-entry atrial flutter in a patient with atrial cardiomyopathy

**DOI:** 10.1093/ehjcr/ytae272

**Published:** 2024-05-29

**Authors:** Sofia Jacinto, Guilherme Portugal, Bruno Valente, Pedro Cunha, Mário Oliveira

**Affiliations:** Arrhythmology, Pacing and Electrophysiology Unit, Cardiology Service, Santa Marta Hospital, Central Lisbon Hospital University Centre, R. de Santa Marta 50, 1169-024 Lisbon, Portugal; Arrhythmology, Pacing and Electrophysiology Unit, Cardiology Service, Santa Marta Hospital, Central Lisbon Hospital University Centre, R. de Santa Marta 50, 1169-024 Lisbon, Portugal; Arrhythmology, Pacing and Electrophysiology Unit, Cardiology Service, Santa Marta Hospital, Central Lisbon Hospital University Centre, R. de Santa Marta 50, 1169-024 Lisbon, Portugal; Arrhythmology, Pacing and Electrophysiology Unit, Cardiology Service, Santa Marta Hospital, Central Lisbon Hospital University Centre, R. de Santa Marta 50, 1169-024 Lisbon, Portugal; Arrhythmology, Pacing and Electrophysiology Unit, Cardiology Service, Santa Marta Hospital, Central Lisbon Hospital University Centre, R. de Santa Marta 50, 1169-024 Lisbon, Portugal

**Keywords:** Ablation, Atrial cardiomyopathy, Atrial tachycardia, Atrial flutter, Case report, Double-wave re-entry, Three-dimensional mapping

## Abstract

**Background:**

Double-wave macrore-entry is a rare mechanism of atrial tachycardia with limited documentation in the literature. We present a three-dimensional documentation of a double-wave ‘typical’ atrial flutter in a patient with extensive atrial cardiomyopathy.

**Case summary:**

A 78-year-old female with a history of atrial cardiomyopathy and dual-chamber pacemaker for sinus node disease presented with palpitations and incessant atrial flutter. Electrophysiological study revealed a regular tachycardia with a cycle length (TCL) of 230 ms, with proximal to distal coronary sinus (CS) activation. Three-dimensional mapping identified two independent wavefronts circulating the cavotricuspid isthmus (CTI), each with a TCL of 460 ms. Cavotricuspid isthmus ablation resulted in conversion into a distinct tachycardia with left atrial roof origin. Linear ablation in this location slowed the TCL to 435 ms with concentric CS activation and another CTI dependent atrial flutter was mapped, this time with only one wavefront of activation. Further ablation with a second, more lateral, line in the CTI led to tachycardia interruption. Given the extensive atrial scarring and high arrhythmic recurrence risk, atrioventricular node ablation was performed.

**Discussion:**

Double-wave re-entrant tachycardias were primarily observed in experimental models, precipitating acceleration of ventricular and supraventricular tachycardias via extrastimulation. In our case, there is documentation of a spontaneous double-wave of activation around the CTI, representing the first documented double-wave ‘typical’ atrial flutter. Unlike other cases in the literature, the two wavefronts were equidistant, which resulted in a regular tachycardia with TCL that was half of the single-wave cycle length. Three-dimensional propagation mapping was essential to visualize the two distinct wavefronts.

**Learning points**

- Double-wave re-entry (DWR) is a rare mechanism of sustained atrial tachycardia, with scarce clinical documentation in the literature.
- Double-wave re-entry has been described in experimental models, whereas an extrastimulus is blocked near the pacing site, not colliding with the subsequent orthodromic wave and generating two waves within the same circuit.
- Three-dimensional mapping of DWR with a propagation map is crucial as a visual aid to display the presence of two distinct wavefronts.
- In this case, we document a spontaneous DWR typical atrial flutter in a patient with atrial cardiomyopathy.
- This case highlights the DWR tachycardia as an uncommon entity that might be anticipated in patients with extensive atrial scarring.

## Introduction

Double-wave macrore-entry is a rare mechanism of atrial tachycardia with few reports in the literature and scarce three-dimensional documentation. In this case, we report on a patient with extensive atrial cardiomyopathy and multiple atrial arrhythmias including a double-wave typical atrial flutter.

## Summary figure

**Figure:**
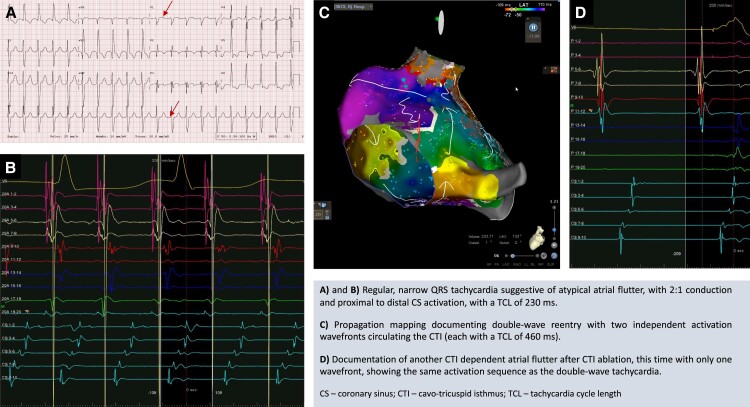


## Case presentation

**Figure 1:**
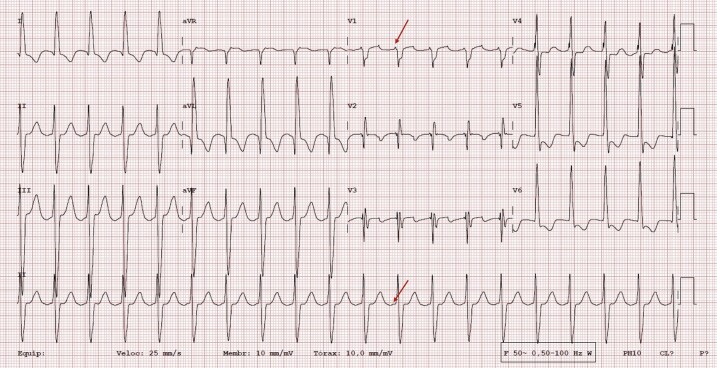
Twelve-lead electrocardiogram of the atrial tachycardia. Note the positive F waves in lead V1 and isoeletric in lead II.

A 78-year-old woman with a history of atrial cardiomyopathy, sinus node disease and long-standing dual-chamber pacemaker presented with palpitations and an incessant atrial tachycardia despite antiarrhythmic therapy, leading to tachycardia induced cardiomyopathy. Atrial cardiomyopathy was diagnosed on the basis of structural and electrophysiological changes such as the presence of sinus node disease, documentation of several previous atrial tachycardias, and significant atrial enlargement. She was medicated with rivaroxaban 20 mg i.d., amiodarone 200 mg i.d., and bisoprolol 5 mg b.i.d. Physical examination was unremarkable. Transthoracic echocardiogram revealed a reduced left ventricular ejection fraction of 40%, global hypokinesia of the left ventricle, and dilation of both atria (left atrium volume of 45 mL/m^2^ and right atrium of 32 mL/m^2^). Twelve-lead electrocardiogram of the tachycardia showed a regular, narrow QRS with a ventricular rate of 120 beats per minute (b.p.m.) and a 2:1 conduction, with positive F waves in lead V1, isoeletric in lead II, suggestive of atypical atrial flutter (*Figure 1*).

**Figure 2:**
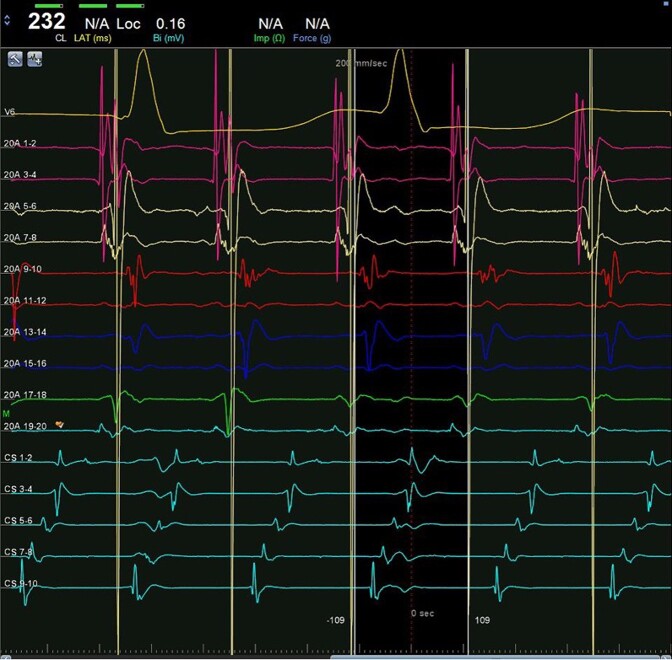
Intracardiac EGM showing a regular atrial tachycardia with 2:1 conduction, proximal to distal coronary sinus activation and a cycle length of 232 ms. PentaRay™ catheter is positioned at 12 o’clock on the tricuspid annulus with activation of Poles 9–10 simultaneous with CS ostium.

**Figure 3:**
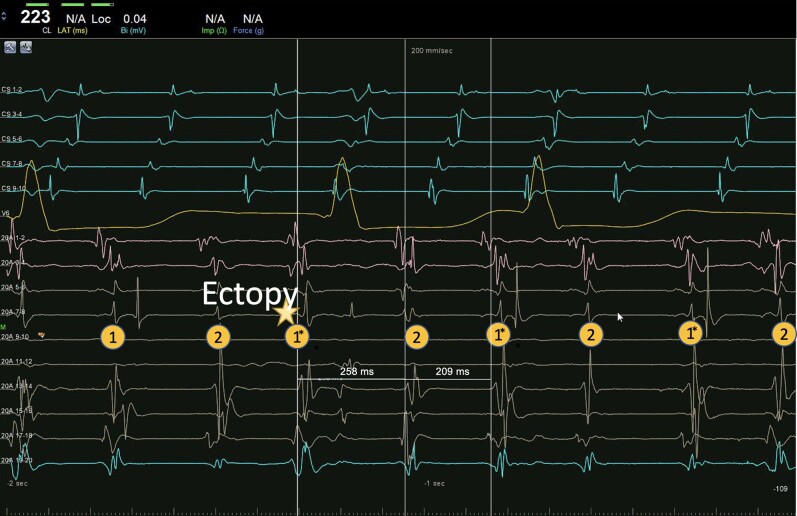
Intracardiac EGM of the double-wave re-entry tachycardia showing variable tachycardia cycle length after an atrial ectopy. EGMs of both waves are marked with 1 or 2. Note the reset of the tachycardia after the ectopy (1*) resulting in a larger interval of 258 ms followed by a shorter interval of 209 ms consistent with double-wave re-entry. The sum of both intervals is 467 ms, which is double the tachycardia cycle length. PentaRay™ catheter is positioned at 12 o’clock on the tricuspid annulus.

Electrophysiological (EP) study was performed after transoesophageal echocardiogram that excluded intracavitary thrombi and identified a patent foramen ovale. Intracardiac electrogram (EGM) showed a regular tachycardia with 2:1 conduction, a tachycardia cycle length (TCL) of 230 ms, and a proximal to distal coronary sinus (CS) activation (*Figure 2*). Right atrium (RA) mapping was performed using CARTO™ (Biosense Webster, Inc.) three-dimensional navigation system and PentaRay™ (Biosense Webster, Inc.) high density catheter. Voltage map showed extensive scarring in the right posterior wall and around the inferior vena cava (see supplementary material online, *Video 1*—right panel). Propagation mapping documented two independent activation wavefronts, circulating the cavotricuspid isthmus (CTI) in a counterclockwise manner. Each wave followed the same circuit at the same time and had a TCL of 460 ms, that is, twice the apparent tachycardia cycle length (see supplementary material online, *Video 1*—left panel). Entrainment manoeuvres were not performed at the risk of terminating the tachycardia. The presence of an atrial ectopy resulted in reset of the tachycardia followed by an alternating cycle length of 258 and 209 ms (*Figure 3*). The sum of both these intervals was double the TCL, suggesting a double-wave mechanism. Cavotricuspid isthmus ablation was performed using Thermocool Smarttouch™ catheter (Biosense Webster, Inc.) with a power setting of 35 W, contact force of 5–30 g, and an Ablation Index™ (Biosense Webster, Inc.) target value of 450 (see supplementary material online, *Figure S1*).

**Figure 4:**
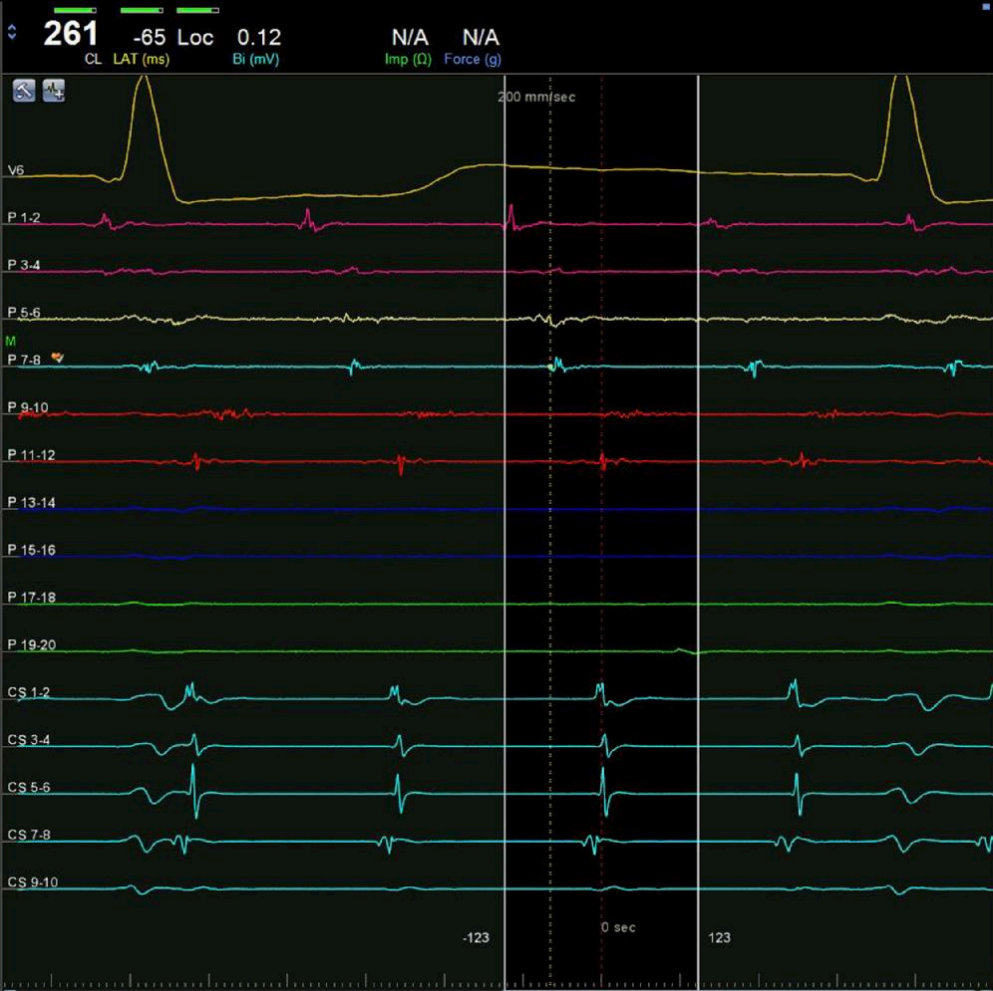
EGM tracing of the atrial tachycardia with simultaneous proximal and distal CS activation with a cycle length of 250 ms, suggestive of left atrial roof origin.

This ablation resulted in conversion of the atrial flutter into a distinct tachycardia with simultaneous proximal and distal CS activation and a cycle length of 250 ms, suggestive of left atrial roof origin (*Figure 4*). Left atrium (LA) access was established via the patent foramen ovale, and mapping was performed with PentaRay™ (Biosense Webster, Inc.) catheter. A complex left atrial macrore-entrant tachycardia with an earliest pseudofocal activation at the proximal insertion of the Marshall ligament, below the left pulmonary veins, was noted. Extensive scarring of the LA was also evident on the voltage map (see supplementary material online, *Video 2*).

**Figure 5:**
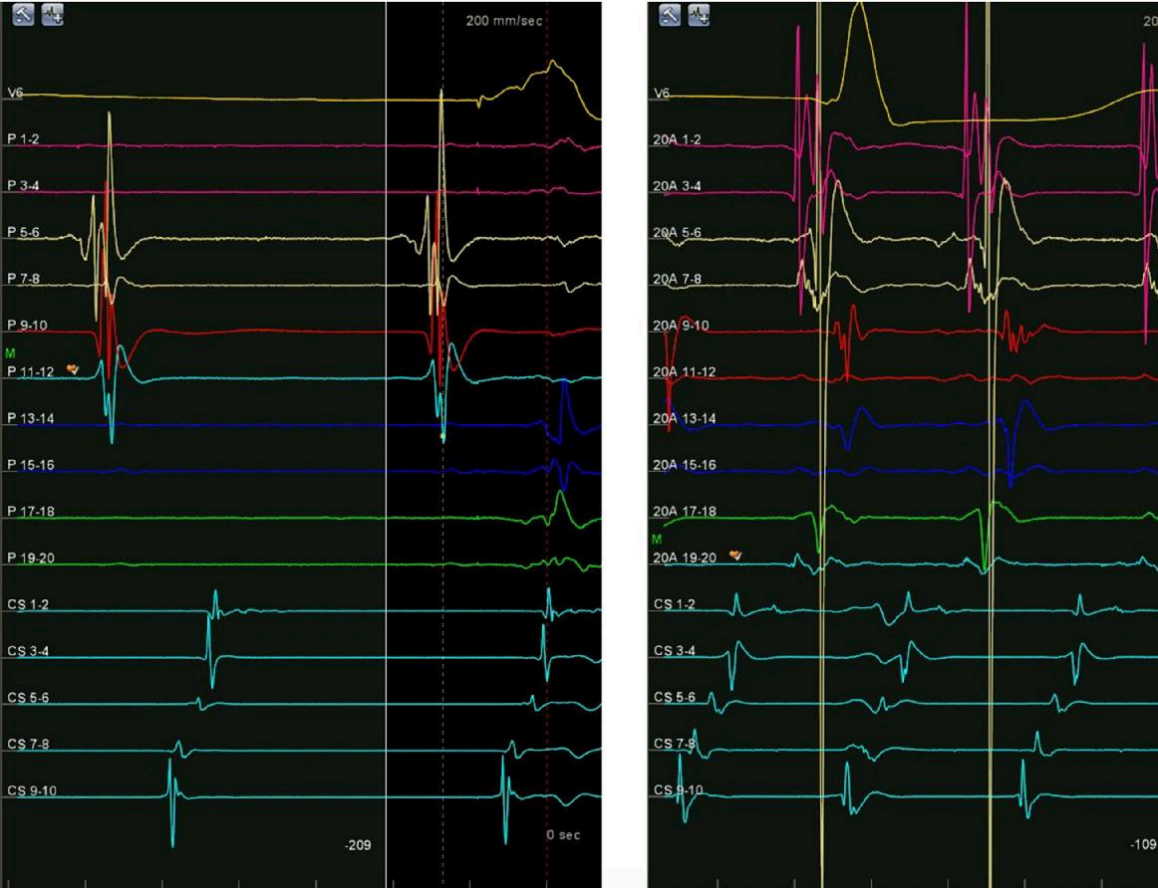
EGM tracings comparing double-wave (right) and single-wave (left) tachycardias, noting the same activation sequence in the CS.

Linear ablation in this location (see supplementary material online, *Figure S2*) led to slowing of TCL to 435 ms with change to concentric CS activation, so a new RA map was obtained. Once again, a CTI dependent atrial flutter with counterclockwise activation was noted, but this time with only one wavefront (see supplementary material online, *Video 3*). Comparison of EGM tracings from double to single-wave showed the same activation sequence in the CS decapolar catheter (*Figure 5*). Further ablation with a second, more lateral, line in the CTI (see supplementary material online, *Figure S3*) led to interruption of the tachycardia. Bidirectional CTI block was confirmed and no tachycardia was induced at the end of the procedure.

Ultimately, given the extensive atrial scarring and high probability of arrhythmic recurrence, atrioventricular (AV) node ablation was performed. The pacemaker was programmed as DDD at 80 b.p.m.

At follow-up 12 months after ablation, the patient has experienced no recurrent palpitations or symptoms of heart failure. Interrogation of the device showed paroxysmal recurrence of atrial high rate episodes that were self-limited.

## Discussion

The occurrence of spontaneous double-wave re-entry tachycardia in humans is a rare phenomenon, with only a limited number of cases documented in the medical literature. This mechanism was initially described by Brugada *et al*.^1^ in 1990, in an animal model, as a cause of acceleration of re-entrant ventricular tachycardia (VT) during programmed electrical stimulation. The proposed explanation is that if an extrastimulus is blocked near the pacing site, it will not collide with the subsequent orthodromic wave, thereby generating a new wave within the circuit. Both waves travel through partially refractory tissues, resulting in a decreased conduction velocity, with the wavelength being shorter than half the size of the circuit. Despite its observation in this study, there remains a lack of clinical demonstrations of spontaneous double-wave re-entry VT.^1^

Regarding supraventricular arrhythmias, Cheng *et al*.^2^ reported double-wave re-entry during typical atrial flutter after delivering a single premature stimulus at the CTI shortly after the end of the refractory period. Similar to VT models, the TCL of the double-wave re-entry was approximately half the TCL of the original flutter. This was confirmed by the simultaneous occurrence of local activation at opposing anatomical sites with identical activation sequences and electrocardiogram morphologies.^2^ More recently, Maury *et al*.^3^ documented a case of double-wave left atrial tachycardia with an irregular cycle length, where the two waves were not evenly situated within the circuit, resulting in an irregular cycle length.

What distinguishes this case is the apparent spontaneity of double-wave tachycardia within the CTI circuit, making it, to our knowledge, the first documented case of double-wave ‘typical’ atrial flutter. Intriguingly, in this case, the two wavefronts were equidistant, creating the impression of a tachycardia with a regular cycle length of 230 ms, in contrast to the irregular cycle length reported by Maury *et al*.^3^

Present three-dimensional electro-anatomical systems may encounter challenges when mapping double-wave re-entry as they tend to register the total cycle length, which is double the true TCL. Therefore, a propagation map becomes crucial as a visual aid to display the presence of two distinct wavefronts. In this case, multiple explanations for visualizing two propagation waves in the same circuit may include errors in mapping settings (such as an incorrect doubling of the window of interest interval), focal activity within the RA leading to pseudo-re-entry due to conduction block near the tachycardia origin, a re-entrant circuit within the LA with passive activation of the RA, or genuine double-wave re-entry. However, both the findings in the propagation map and electrograms substantiate the hypothesis of double-wave re-entry. Reset of the tachycardia after an ectopy suggests a re-entrant mechanism. In the case of a single-wave re-entry, an ectopy resetting a tachycardia should have a post-pacing interval equal to the TCL. However, in the case of a dual-wave re-entry, this would lead to first a larger and then a smaller interval, with the sum of both equal to double the TCL. The simultaneous focal activation of two anatomical sites with identical activation sequences and electrocardiogram morphology, as observed during spontaneous typical flutter, strongly suggests a double-wave mechanism.

Medical treatment of double-wave re-entry tachycardia may be aided by the use of class III antiarrhythmic drugs (AAD) as suggested by Brugada *et al*.^1^ In their animal model, the use of a class III AAD decreased the excitable gap of the VT and double-wave re-entry induction was no longer possible. Nevertheless, clinical evidence is still lacking.

Furthermore, this case underscores the significance of recognizing electrical disturbances associated with atrial cardiomyopathy,^4^ ultimately culminating in the need for AV node ablation, especially in a patient with a pre-existing implanted pacemaker, to attain symptomatic control. This highlights the intricate interplay between structural and electrical factors in arrhythmia management.

## Supplementary Material

ytae272_Supplementary_Data

## Data Availability

The data underlying this article are available in the article and in its online Supplementary material.

## Lead author biography

Dr Sofia Jacinto is a Cardiology resident from Lisbon, Portugal. She graduated from NOVA Medical School and has spent the last five years working in Santa Marta Hospital, a central university hospital in Lisbon. Currently, Dr Jacinto is embarking on her electrophysiology and cardiac implantable electronic devices (CEID) training in the Arrhythmology Unit. She is also collaborating in the Cardiology remote monitoring clinic, working with pioneering risk stratification algorithms for patients with CIED. Her record also encompasses scientific presentations at national and international congresses and co-investigator roles in clinical trials.

## Supplementary material

**Consent:** The authors confirm that the written consent for submission and publication of this case report, including images and associated text, has been obtained from the patient in line with COPE guidance.

**Funding:** The authors have not declared a specific grant for this research from any funding agency in the public commercial or not-for-profit sectors.
