# Supplementary figures and images for: Case report of a double-wave re-entry atrial flutter in a patient with atrial cardiomyopathy
Source: Eur Heart J Case Rep. 2024 May 29;8(6):ytae272. doi: 10.1093/ehjcr/ytae272 (PMC11165273; doi:10.1093/ehjcr/ytae272)

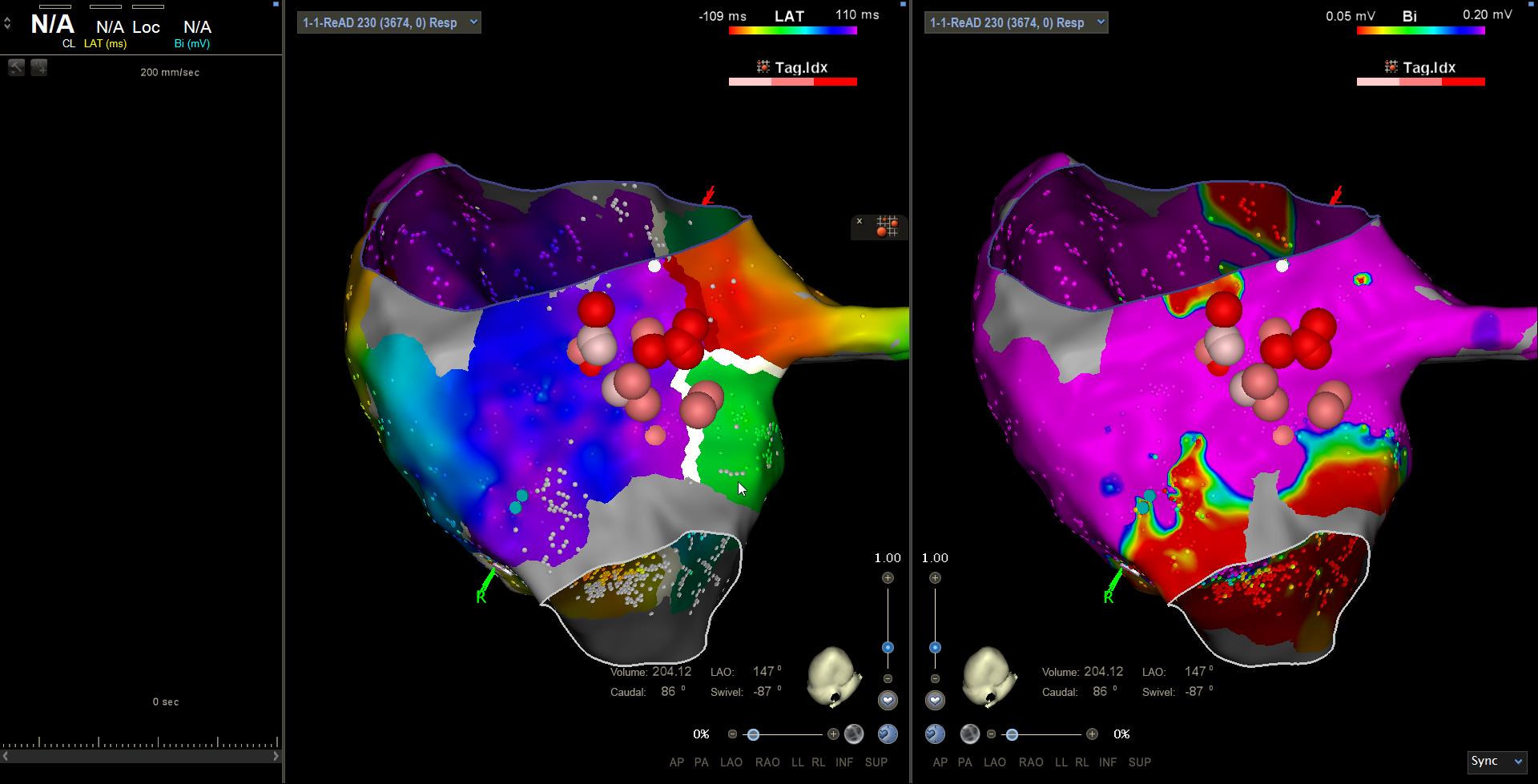

Supplement: ytae272_Supplementary_Data [file ytae272_supplementary_data.zip › Supplementary figure 1.jpg]

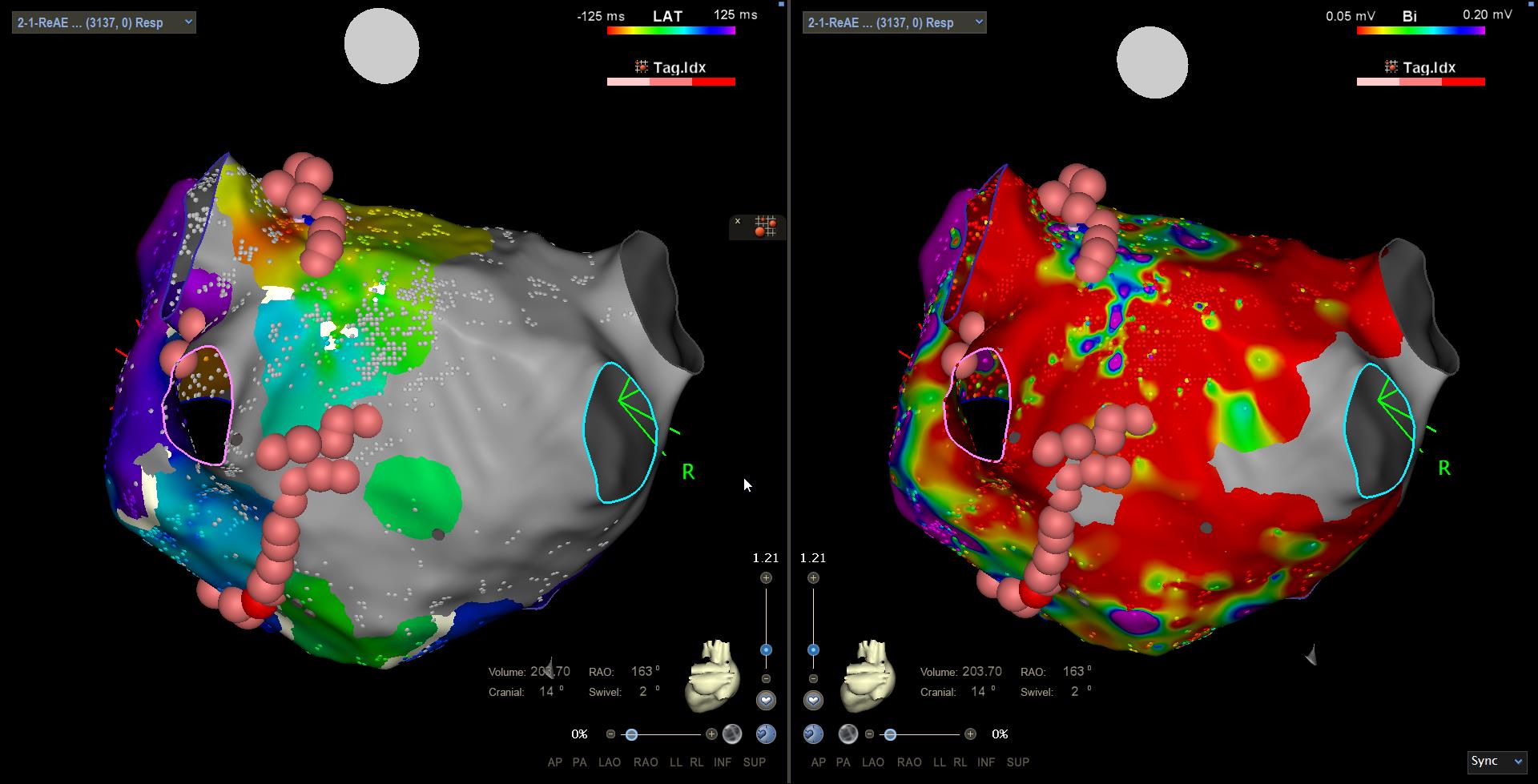

Supplement: ytae272_Supplementary_Data [file ytae272_supplementary_data.zip › Supplementary figure 2.jpg]

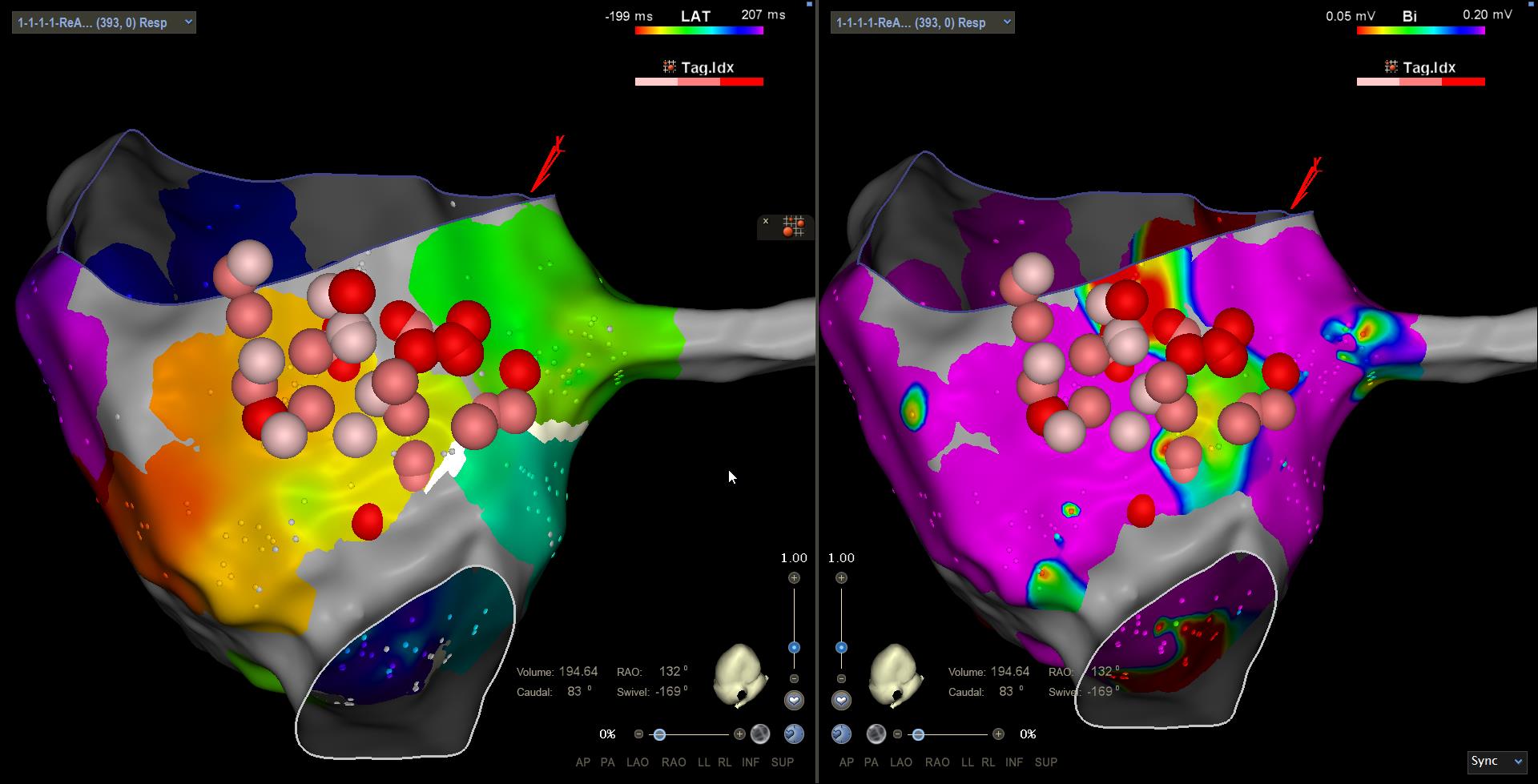

Supplement: ytae272_Supplementary_Data [file ytae272_supplementary_data.zip › Supplementary figure 3.jpg]
